# Domestic Dogs and Wild Foxes Interactions in a Wildlife-Domestic Interface of North-Central Chile: Implications for Multi-Host Pathogen Transmission

**DOI:** 10.3389/fvets.2021.631788

**Published:** 2021-02-09

**Authors:** Felipe A. Hernández, Jonatan Manqui, Carlos Mejías, Gerardo Acosta-Jamett

**Affiliations:** ^1^Instituto de Medicina Preventiva Veterinaria, Facultad de Ciencias Veterinarias, Universidad Austral de Chile, Valdivia, Chile; ^2^Programa de Magíster en Ecología Aplicada, Facultad de Ciencias, Universidad Austral de Chile, Valdivia, Chile; ^3^Instituto de Ciencias Ambientales y Evolutivas, Facultad de Ciencias, Universidad Austral de Chile, Valdivia, Chile; ^4^Programa de Investigación Aplicada en Fauna Silvestre, Facultad de Ciencias Veterinarias, Universidad Austral de Chile, Valdivia, Chile

**Keywords:** domestic dogs, wild foxes, camera trapping, interface, interactions, pathogens, Chile

## Abstract

Domestic dogs (*Canis familiaris*) often cohabite at interfaces shared by humans and wildlife, interacting with wild canids as predators, prey, competitors and reservoirs of several multi-host pathogens, such as canid-borne micro and macro parasites that could impact on wildlife, livestock and public health. However, spatio-temporal patterns of indirect interactions as promoters of pathogen transfer between domestic and wild canids are largely unknown. In this study, we used camera traps to describe the activity patterns and habitat use of dogs, chilla (*Lycalopex griseus*) and culpeo (*Lycalopex culpaeus*) foxes and identify the local-scale factors that may affect the frequency of dog-fox interactions through an anthropization gradient of the Coquimbo region, Chile. We assessed local-scale variables that may predict the number of interactions between dogs and foxes, and compared the time interval between dog-culpeo and dog-chilla interactions. Our findings suggested that closeness to urbanized zones predicts the frequency of indirect interactions between dogs and foxes. We found higher number of dog-fox interactions (60 interactions) at a periurban site adjacent to two coastal towns (Tongoy and Guanaqueros), compared to other two more undisturbed sites (12 interactions) increasingly distanced from urbanized areas. We showed that dogs interacted more frequently with chilla foxes (57 interactions) than with culpeo foxes (15 interactions), and the first interaction type occurred almost exclusively at the periurban site, where dogs and chillas were more frequently detected than in the other sites. We detected a marked temporal segregation between dogs and foxes, but dog-chilla interactions resulted in shorter time intervals (2.5 median days) compared to dog-culpeo interactions (7.6 median days), suggesting a higher potential risk of pathogen spillover between the first species pairing. Based on previous studies, we suggest periurban zones may constitute a potential focus of pathogen exposure between dog and fox populations in the study area. Our research contributes to improving the knowledge on the spatio-temporal patterns of interspecific contact between invasive and native carnivores within the context of multi-host pathogen dynamics. Our outcomes will inform theoretical epidemiological models designed to predict and minimize the contact risk between domestic and threatened species, guiding effective control strategies at the wildlife-domestic interface.

## Introduction

Interspecific interactions are relevant behavioral factors that modulate population and community dynamics at multiple ecological levels (1, 2). From an epidemiological perspective, there are direct and indirect interactions that could facilitate pathogen transmission. Direct interactions involve spatial and temporal co-occurrence and often a specific behavioral interaction, while indirect interactions only require spatial co-occurrence of reservoir and susceptible hosts within a certain time window (3). The second may determine indirect cross-species transmission of several environmentally-resistant pathogens (including viruses, bacteria, and macro parasites), where the interacting hosts would be exposed to the contaminated environments they share (4–6).

Created by the encroachment of human activities into natural landscapes, wildlife-domestic interfaces represent critical boundaries where humans, vectors, and reservoir hosts (wildlife or domestic animals) coexist, thus increasing contact and the risk of cross-species transmission and emergence of pathogens (7–9). During the last few decades, several hypotheses about how wildlife-domestic animals interactions can contribute to infectious disease emergence have been tested. For example, relatively frequent opportunities of indirect contact between domestic and wild ungulates at shared water and food points have showed to play a relevant role in the transmission of pathogens of economic and public health concern, including agents of bovine tuberculosis, brucellosis, pseudorabies, and African swine fever (10–15), among others. However, there have been relatively few empirical attempts to quantify local-scale spatio-temporal patterns of indirect contact within other mammalian groups, such as carnivores [e.g., (16, 17)] in the context of the potential impact that invasive species may have on pathogen transmission for vulnerable wildlife.

As invasive species expand globally, further research is needed to understand the interspecific interactions between native and non-native carnivores, considering the risk posed by persistently infected invader hosts in promoting the global spread of pathogens into naïve native wildlife (18). Domestic dogs (*Canis familiaris*) is one of the most ubiquitous and damaging invasive carnivore worldwide, often cohabiting at the interface between human and wildlife communities (19–21). Most regions of the developing world harbor large, mostly unvaccinated and rarely dewormed dog populations, regularly allowed to roam freely, which can potentially interact with wildlife as predators, prey, competitors and reservoirs of pathogens (20, 22, 23). Domestic dogs can act as pivotal “bridge hosts” of infectious diseases and serve as a source of several multi-host pathogens with important consequences for wildlife conservation and animal and public health (24, 25). For instance, contact between domestic dogs and wild carnivores can facilitate transmission of micro parasites [e.g., canine distemper virus (CDV) and canine parvovirus (CPV)] or macro parasites (e.g., endoparasites, ticks, fleas, among others) (26–31). Conversely, wild canids can also be an important source of infection to domestic animals and humans at transition zones between wilderness and urban areas (23). As an example, the greater abundance of some wild animals, as it has occurred with red foxes in Europe, correlates with an increase in risk of *Echinococcus multilocularis* transmission to humans in periurban and urban areas (27).

Studies in Chile focused on dog demography and the risk of pathogen spillover from dogs to wildlife and vice versa have reported higher dog densities in urban than rural areas (32, 33), with urbanized zones constituting a critical source of micro and macro parasites capable to jump into locally and regionally common wild canid species, such as chilla (*Lycalopex griseus*) and culpeo (*Lycalopex culpaeus*) foxes (33–36). In the semiarid Coquimbo region of north-central Chile, a higher likelihood of CDV and CPV exposure was estimated for chilla and culpeo foxes inhabiting in proximity to urban compared to rural areas (34, 35), with chillas thriving more frequently within human-perturbed landscapes (34, 35). However, the spatio-temporal patterns of interspecific interactions that may be causing pathogen spread between domestic and wild canids are largely unknown in Coquimbo, a zone where a CDV epidemic struck specimens of both fox species in 2003 (37), and where domestic dogs are highly parasitized by ticks and fleas and possible vector-borne transmission between domestic and wild canids can occur (36, 38).

Camera traps constitute an increasingly popular non-invasive and cost-effective monitoring tool for cryptic and crepuscular species (39, 40), allowing the detection and quantification of contact rates between a range of invasive (including dogs) and native carnivores within the context of pathogen spillover risk [e.g., (24, 41–43)]. In this study, we used camera traps to describe the activity patterns and habitat use of dogs, chilla and culpeo foxes and identify the local-scale factors that may affect the frequency of dog-fox interactions trough a wildlife-domestic interface of the Coquimbo region. Our study refers exclusively to interspecies interactions (not a pathogen transmission model), and thus, their potential role in facilitating canid-borne pathogen transfer between domestic and wild canids. First, we estimated the detection rates of dogs and foxes to describe changes in the intensity of habitat use of domestic and wild canids over selected sites conforming an anthropization gradient from urbanized to rural zones. Second, as a behavioral mechanism of interspecies coexistence, we hypothesized that temporal activity patterns displayed by dogs and foxes will differ across all sites. Third, assuming that rural interfaces nearby urbanized areas facilitate interactions between domestic and wild canids, we tested the hypothesis that periurban areas in proximity to human settlements and main roads will support higher number of indirect interactions between dense dog populations and foxes compared to rural areas more distanced from towns. Finally, assuming chilla foxes would be more tolerant to thrive into human-dominated landscapes, we hypothesized that we would find shorter time intervals between dog and chilla visits compared to dog and culpeo visits.

## Materials and Methods

### Study Area

**Figure 1:**
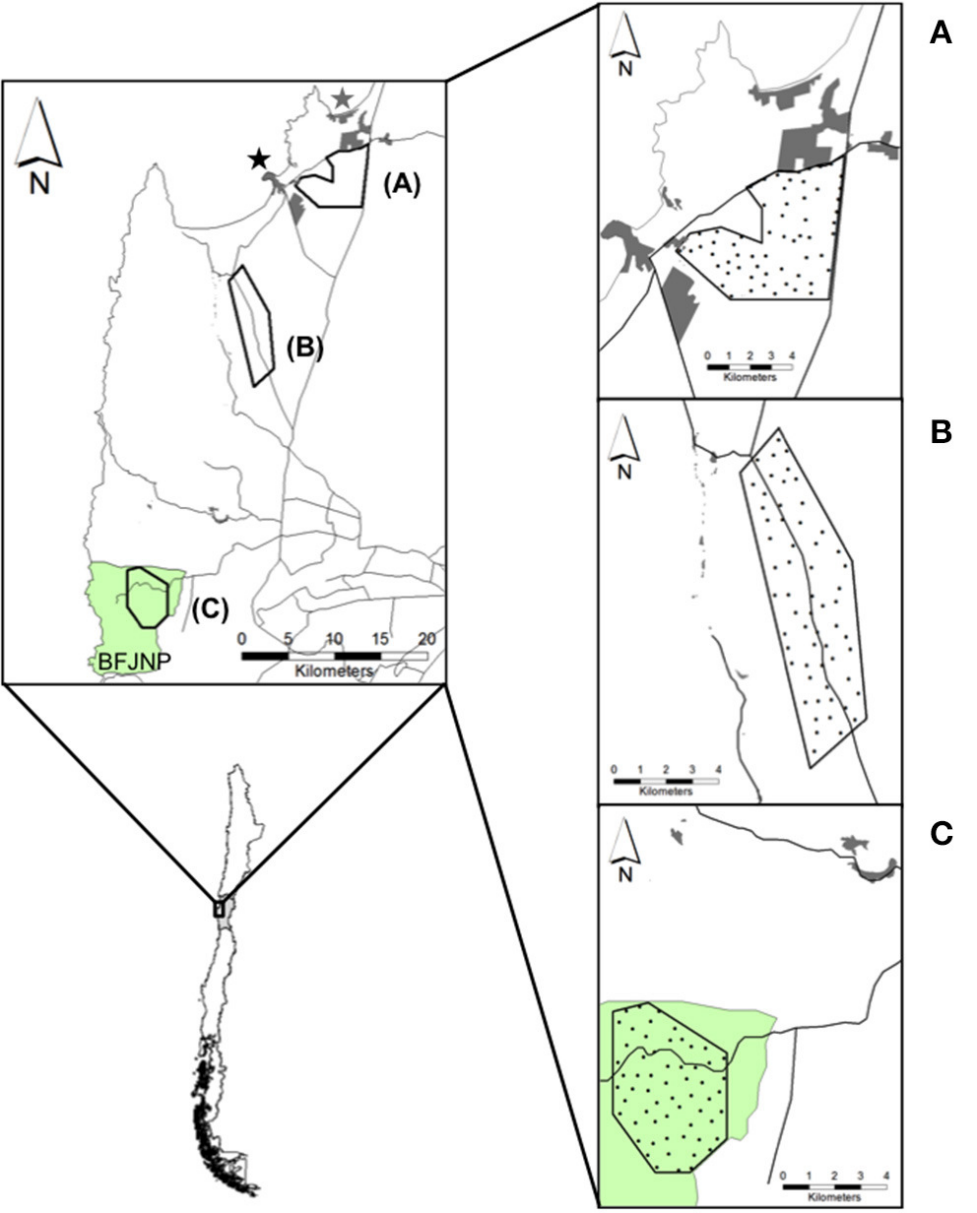
Map of field sites for camera trap monitoring in the coastal zone of the Coquimbo region, Chile. Top left: **(A)** Periurban, **(B)** Rural, and **(C)** Wild sites. Tongoy (black star) and Guanaqueros (gray star) towns, and Bosque Fray Jorge National Park (BFJNP; green area) are indicated. Right: Black circles over each site indicate camera trap stations. Proximate human settlements (gray polygons) and main roads (black lines) are drawn around each site.

The study was conducted in the coastal zone of the Coquimbo region in north-central Chile (71°12′ to 71°40′W, 29°58′ to 30°39′S) (Figure 1). The study area poses a semiarid Mediterranean weather with a mean annual rainfall of 126.8 mm, with 90% of rainfall concentrated during winter months (May–September), and warm, dry summers (December–March) (44, 45). Mean temperature ranges from 12 to 18°C (measured at 2 m above ground nearby the coast), and relative air humidity can reach 90 to 100% at higher altitudes.

The landscape is a mosaic of native vegetation and human land use covers (46) characterized by a system of coastal towns and inland villages, rural communities with agricultural and livestock lands, and a state protected area, which as a whole compound a contiguous gradient of urbanization running from north to south. During the last decade, increased land-use change caused by settling of villages, neighborhoods and land subdivision for household construction have rapidly evolved around two towns in the area, Tongoy and Guanaqueros towns, and the road network connecting both urban centers (including the Pan-American Highway), resulting in wildlife-domestic interfaces were rural and urban habitats intersect (Figure 1). Rural areas are conformed by agricultural fields, pastures, and a mixture of exotic shrub plantations (i.e., *Atriplex nummularia*) and pioneer native vegetation. Rural economy has been historically based on livestock production, mostly focused on small ruminant farming (47). The only protected area in the zone corresponds to the Bosque Fray Jorge National Park (hereafter BFJNP), a ca. 10,000 ha World Biosphere Reserve located at the coastal, northern edge of the Chilean Mediterranean region, which protects diverse semiarid ecosystems and relict forests of the Coastal mountain range. BFJNP is dominated by thorn scrub, cactus and other succulent plants (48).

### Study Design

In our study area, we defined three field sites running from Tongoy and Guanaqueros suburbs through areas of decreasing human perturbation to BFJNP (Figure 1). Sites were characterized as follows: (1) Periurban, a private 33 km^2^-site characterized by shrubby open habitat dominated by *A. nummularia* and ruderal native plants located in close proximity to urban and suburban areas (1.5 km east of Tongoy); (2) Rural, a private 31 km^2^-site inserted into the “El Tangue” ranch, a farm of nearly 45,000 ha situated 10 km south of Tongoy. The property is characterized by a mosaic of agricultural fields, grasslands, and a mixture of exotic and native shrubs, with small properties scattered along the westward border distance up to 3 km of the field site; and (3) Wild, a 22 km^2^-site characterized by a xeric-mesic ecosystem into the BFJNP, located at 43 km south of Tongoy. Geographical distancing across sites was considered to ensure independence of dog-fox interactions and sites were arbitrarily chosen based on the hypothesis of higher opportunities of dog-fox interactions and pathogen spillover in proximity to urban zones compared to rural and undisturbed zones. High dog densities have been reported across towns in the Coquimbo region [i.e., ~1,500 dog/km^2^; (32)], which may increase encounters with wild carnivores. While both the rural and wild sites harbor lower dog densities compared to urbanized zones [i.e., ~2 dog/km^2^; (32)], decreased opportunities of encounters between dogs and foxes would be expected. Compared to dogs, wild foxes are distributed at lower densities, reaching nearly 0.5 fox/km^2^ as reported for pre-Andean zones in the Coquimbo region (49).

### Camera Trap Survey

To determine activity pattern and dog-wild canid interactions, at each site we randomly selected 60 camera stations separated by a minimum distance of 500 m using the ArcGIS® program. At each station we deployed an infrared camera trap (Bushnell Trophy Cam®, Bushnell Corporation, Overland Park, KS, USA) during two sampling periods, August 2018–April 2019 (year 1) and August 2019–February 2020 (year 2). Once we visited the sites, we recorded GPS location for each camera in UTM coordinates using WGS84 projection. Cameras were mounted on wood poles ca. 50–60 cm above the ground on the side with the more open detection zone, facing toward either south or south-west direction to minimize overexposure by sun light. To avoid perturbing animal detection, we only cleared leaves or branches that were within 1 m of the camera lens. No olfactory bait was used at camera stations to ensure that we detected natural canid behavior. We programmed each camera to shoot three pictures per trigger with a 30-min delay between consecutive triggers (to minimize shooting of empty photos). All cameras were installed and activated simultaneously in one particular site, and after a minimum of 20 days, they were retrieved and reinstalled on a next site. Depending on work schedule conflicts, timing of camera retrieval occasionally varied across sites. Sampling scheme was repeated during two consecutive seasons per year (i.e., austral spring and summer), totaling four sampling seasons per site. Photos were systematically backed up between seasons and proper camera functioning was checked.

### Metadata Management and Species Identification

To conduct a systematic data management, each photo was associated with metadata including unique identification code, species, date and time of photo-capture day. We contrasted field dates of camera installment and retirement with the meta-information stored at each photo. We corrected any data discrepancy using the command-line application ExifTool v.11.56 (50), and renamed all photos according to their corresponding site, date and time using the package “camtrapR” (51) under R (52). We selected and tagged photos using the application digikam v.6.1.0 (KDE applications, Berlin, Germany). For identification of canid species, we relied on distinctive morphological traits that characterize either dogs, culpeo or chilla foxes. Photos with more than one animal in the frame were counted and tagged as one-detection for the corresponding species. When photos were unable to be readily identified as a particular species (e.g., due to poor image quality or partial body detection), they were tagged as “unknown species” and not included in the analyses.

### Data Analyses

For their inclusion in the analyses, we considered a 30-min interval as a measure of independence between photos of the same species taken by the same camera, following Bitetti et al. (53). The number of days in which each camera was active and able to be triggered was denoted as camera trap-nights (CTN). We estimated the trapping success (number of independent photos/CTN × 100 trap-nights) as an indicator of intensity of habitat use exhibited by each species per site, following Burton et al. (40). Total number of cameras and CTN varied across sites, because camera and battery malfunctions and occasional theft of cameras.

#### Quantification and Spatial Description of Dog-Fox Interactions

We quantified the frequency of dog and fox visits to camera stations as proxy of contact rate between species. We defined an indirect interspecies interaction as visit of one species to a camera immediately following the visit of another species within the entire period when the camera was active. We did not restrict the definition of indirect interaction to a shorter time window due to the variable survival times that canid-borne pathogens may have [e.g., CDV ~2 days (54); CPV months to years (55)]. We used the time and date printed at each photo to estimate the time interval between visits of dogs and foxes. To account for bi-directional contact rates which may potentially facilitate pathogen spillover or spillback between domestic and wild canids, we calculated the interaction counts, and the mean, median, and range of days between visits of dogs and foxes regardless of the sequence of photos that compound each interaction event (i.e., considering sequences of dog visits followed by fox visits to the same camera, and vice versa). We considered pooling all interaction events as a conservative criterion since the dynamics of ecological interactions among both canid groups and molecular genetic signatures of circulating canid-borne pathogens are largely unknown yet.

Previous studies have used density maps to visualize the density of animal detections and interspecific interactions in a determined neighborhood around those events, contributing to hypothesize about the potential local-scale factors leading to event aggregations over the space [e.g., (12, 42)]. To obtain a preliminary description of the spatial concentration and distribution of dog, fox and interaction occurrences, we obtained a generalized density map over the three field sites. For this purpose, we performed kernel density analyses with the ArcGIS® Spatial Analyst extension, calculating raster surfaces of smoothed density values of the number of culpeo, chilla, dog and interaction events per camera over a gridded surface (10-m grid cell size) within a kernel filter. We calculated the standard distance (i.e., indicator of compactness of camera distribution around the mean center) for all camera stations per site using the ArcGIS® Spatial Statistics toolbox. To define the search radius within which to calculate densities around each camera, we rely on standard distance of cameras to calculate the optimal bandwidth function (56):

$$\begin{array}{l} {\text{h}}_{\text{opt}}=\;{\left[2/3\text{n}\right]}^{\left(1/4\right)}\sigma , \end{array}$$

where *n* is the number of camera stations per site and σ is the standard distance. Previously, depending on the raster surface to create, we weighted the standard distance by either the number of culpeo, chilla, dog or interaction events captured per camera at each site.

#### Activity Patterns

We used a non-parametric kernel density estimation to estimate the probability density function of fox and dog activity patterns using the R-package “overlap” (57). We pooled and compared the activity patterns displayed by dogs and foxes (combining both fox species) at each site using the overlap coefficients Δ_1_ and Δ_4_, which ranges from 0 (no overlap) to 1 (complete overlap), which indicate whether dog-fox pairings overlap in their use of diel cycle periods (i.e., dawn, dusk, day, night). Coefficients Δ_1_ and Δ_4_ have been reported to perform appropriately if the sample has <50 and >75 photos, respectively. The precision of both coefficients was determined by the calculation of confidence intervals as percentile intervals from 1,000 bootstrap samples (58). We conducted a Watson's non-parametric two-sample *U*^2^-test of homogeneity to assess whether dog and fox temporal activities were statistically different using the R-package “circular” (59).

#### Predictors of Dog-Fox Interactions

To assess the local-scale variables that may affect the number of interactions between dogs and culpeo or chilla foxes, we considered five environmental variables corresponding to: (a) site (as previously defined as periurban, rural and wild); (b) year; (c) season; (d) distance from each camera to the nearest known inhabited house, village or city (hereafter human settlements); (e) distance from each camera to the nearest main road; and (f) an indicator of seasonal productivity index (see below). Distances to human settlements and roads were calculated based on human made features within a 2-km buffer around each sampling site, based on the mean maximum distance of foray activity exhibited by dogs from households according to Sepúlveda et al. (60). Spatial data on settlements and roads were obtained from satellite images available in Google Earth. As a surrogate of productivity index, we extracted satellite-derived normalized difference vegetation index (hereafter NDVI, an estimator of vegetation biomass) per each camera seasonally, by obtaining different spectral indexes from the Sentinel-2 satellite platform using the R-package “sen2R” (61). Previously, we created shapefiles in ArcGIS® to represent the study area extension polygons using the “gdal” geospatial toolbox. We obtained a series of satellite images with NDVI values for all sampling seasons over the whole study area, considering a maximum cloudiness of 20–25% and a 10 m spatial resolution. We derived mean NDVI values (range from −1 to +1) within a 250-m buffer around each camera station using the R-packages “raster” (62) and “rgdal” (63). High positive values correspond to vegetation cover that is actively growing, while negative values are typically associated with bare soil or non-vegetated surfaces. Previous studies have demonstrated that higher vegetation biomass (i.e., high positive NDVI values) indicate higher native prey abundance for carnivores [e.g., (64, 65)]. Considering that during seasonal droughts (i.e., a characteristic pattern of semiarid ecosystems in north-central Chile) plant cover and small mammal abundance decrease, foxes could shift toward alternative prey near human settlements (66, 67), enhancing its contact opportunities with dogs.

Given indirect interactions counts between dogs and foxes were over-dispersed (previously tested by using the sum squared Pearson residuals divided by residuals degrees of freedom), we specified models with a negative binomial distribution for the following statistical analyses. First, we used generalized linear mixed-effects models to test whether the number of indirect interactions between dogs and foxes was related to the defined environmental variables. Because cameras sampled repeatedly the same locations during different seasons, we included the camera ID as random effect in all the models. To account for variations in trapping effort across cameras, we included the log of CTN at each camera as an offset variable in all the models (24, 42, 68). We carried out the models using the glmer.nb function from the R-package lme4 (69). For model selection we computed and ranked models by AIC criteria corrected for small sample size (AICc) using the R-package MuMIn (70) and reported the incidence rate ratio (IRR) for each predictor in the most supported models with ΔAICc ≤ 2 (71). Prior to their inclusion in the models, predictor variables were tested for collinearity using Pearson correlation coefficients, and we found no terms exceeding the 0.7 threshold (72). Second, we conducted a Mann-Whitney *U*-test to assess whether the time interval of dog-fox interactions differed between types of interaction (i.e., whether interaction occurred between dog-culpeo or dog-chilla). All the statistical tests used α = 0.05 for determination of statistical significance.

## Results

**Figure 2:**
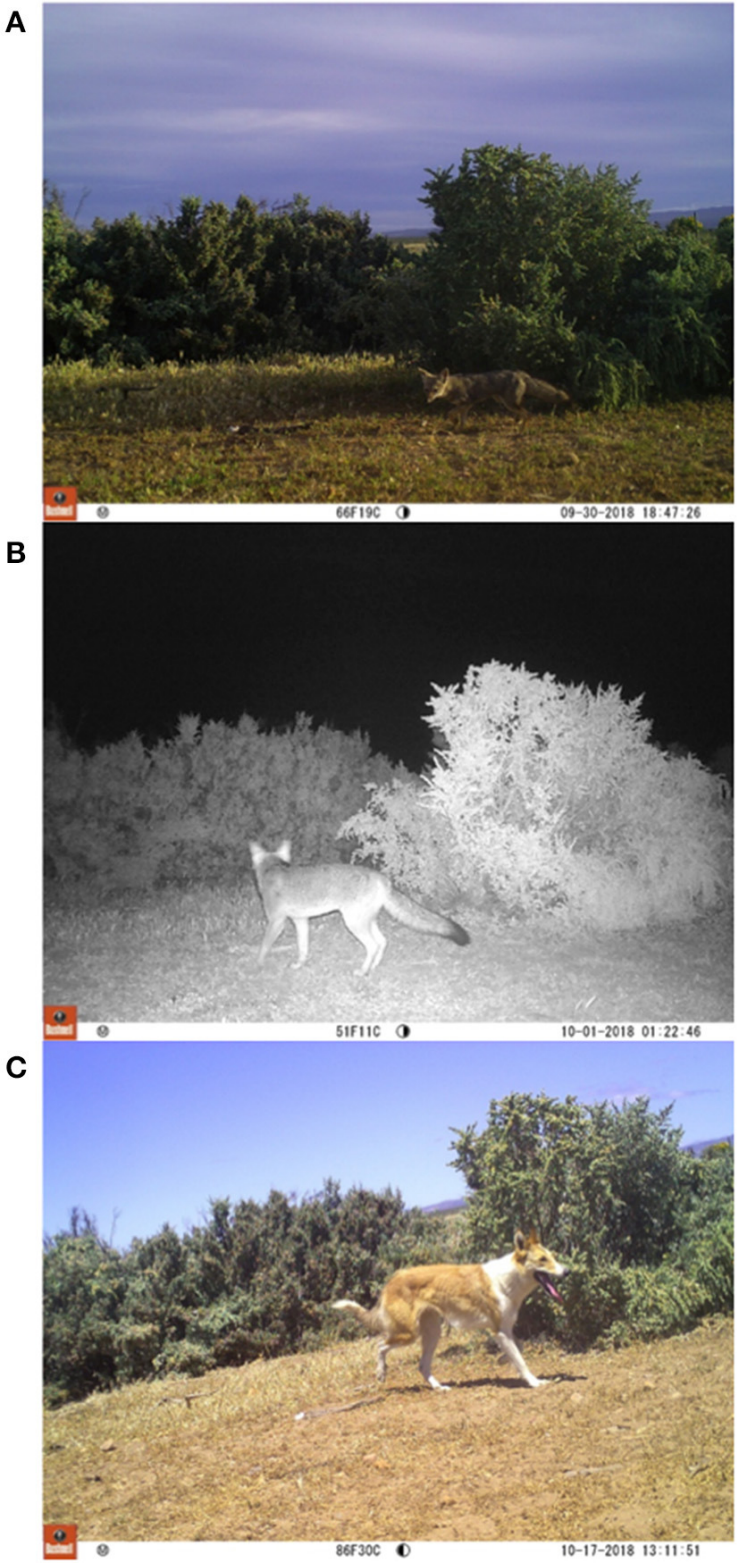
Photos of **(A)** chilla fox, **(B)** culpeo fox, and **(C)** domestic dog recorded at one camera trap during the same season.

Our study produced a total of 2,161 independent photos (hereafter photos) of wild and domestic canids over a total 16,979 CTN (mean 24 ± 0.3 nights per camera, range 1–36 nights per camera). The culpeo fox was the most frequently detected canid species with 1,426 photos, followed by the chilla fox (626 photos) and the domestic dog (109 photos) through the entire study (Table 1 and Figure 2).

**Table 1:** Number of camera stations, camera trap nights (CTN), number of culpeo fox, chilla fox and domestic dog photos, and monitored area per field site in the coastal zone of the Coquimbo region, Chile (August 2018–February 2020).

**Site**	**No. camera stations**	**CTN**	**No. photos**			**Monitored area (km^**2**^)**
			**Culpeo fox**	**Chilla fox**	**Domestic dog**	
Periurban	60	5,380	29	451	91	33
Rural	60	4,923	266	175	14	31
Wild	60	6,676	1,131	0	4	22

**Figure 3:**
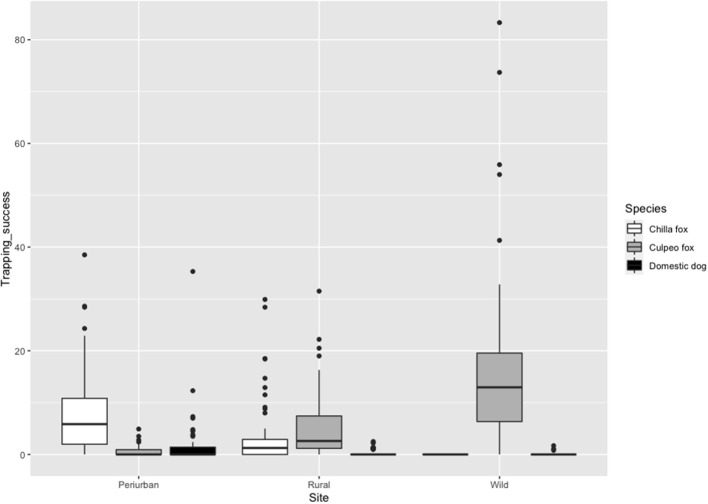
Trapping success (No independent photos/Camera trap nights (CTN) × 100 trap nights) of chilla fox, culpeo fox and domestic dog per field site in austral spring and summer between 2018 and 2020. The darker line represents the median, the colored box includes 50% of data, and the black circles depict the outliers.

Culpeo fox was more frequently detected in the wild site compared to the other sites, resulting in a trapping success three times higher than in the rural site (i.e., 16.9 vs. 5.4 photos/100 CTN, respectively). We totaled 0.5 culpeo's photos/100 CTN in the periurban site. On the contrary, chilla fox displayed a higher intensity of habitat use at the periurban site, with a trapping success more than double than in the rural site (i.e., 8.4 vs. 3.6 photos/100 CTN, respectively). We did not detect chillas in the wild site. Dogs were more frequent at the periurban site, with a trapping success almost six times higher than in the rural site (i.e., 1.7 vs. 0.3 photos/100 CTN, respectively). We only obtained 0.1 dog's photos/100 CTN in the wild site (Figure 3).

### Quantification and Spatial Description of Dog-Fox Interactions

During the entire study, we recorded a total of 72 indirect interactions between dogs and foxes (range: 0–0.2 interactions per trap-night), which were documented by 19, 6, and 3 cameras deployed over the periurban, rural and wild sites, respectively. Dog-chilla fox interactions (*n* = 57) were more frequently recorded than dog-culpeo fox interactions (*n* = 15). In the periurban site, we totaled 60 interactions (55 dog-chilla and 5 dog-culpeo). In the rural site, we detected 9 interactions (7 dog-culpeo and 2 dog-chilla), while we found only 3 dog-culpeo interactions in the wild site (Table 2). We did not reported evidence of potential direct interactions between dogs and foxes (i.e., animals of different species captured in the same photo).

**Table 2:** Number of indirect interactions and time intervals of interactions between dogs and foxes at each field site in the coastal zone of the Coquimbo region, Chile (August 2018–February 2020).

	**Periurban**			**Rural**			**Wild**
	**Dog-chilla**	**Dog-culpeo**	**Total**	**Dog-chilla**	**Dog-culpeo**	**Total**	**Dog-culpeo**
No interactions	55	5	60	2	7	9	3
Average (days)	4.4	8.5	4.8	2.5	7.1	6.1	7.7
Median (days)	2.5	8.0	2.6	2.5	7.6	4.7	3.6
Range (days)	0.1–20.2	0.3–19.8	0.1–20.2	0.3–4.7	0.6–14.7	0.3–14.7	1.5–18.1

**Figure 4:**
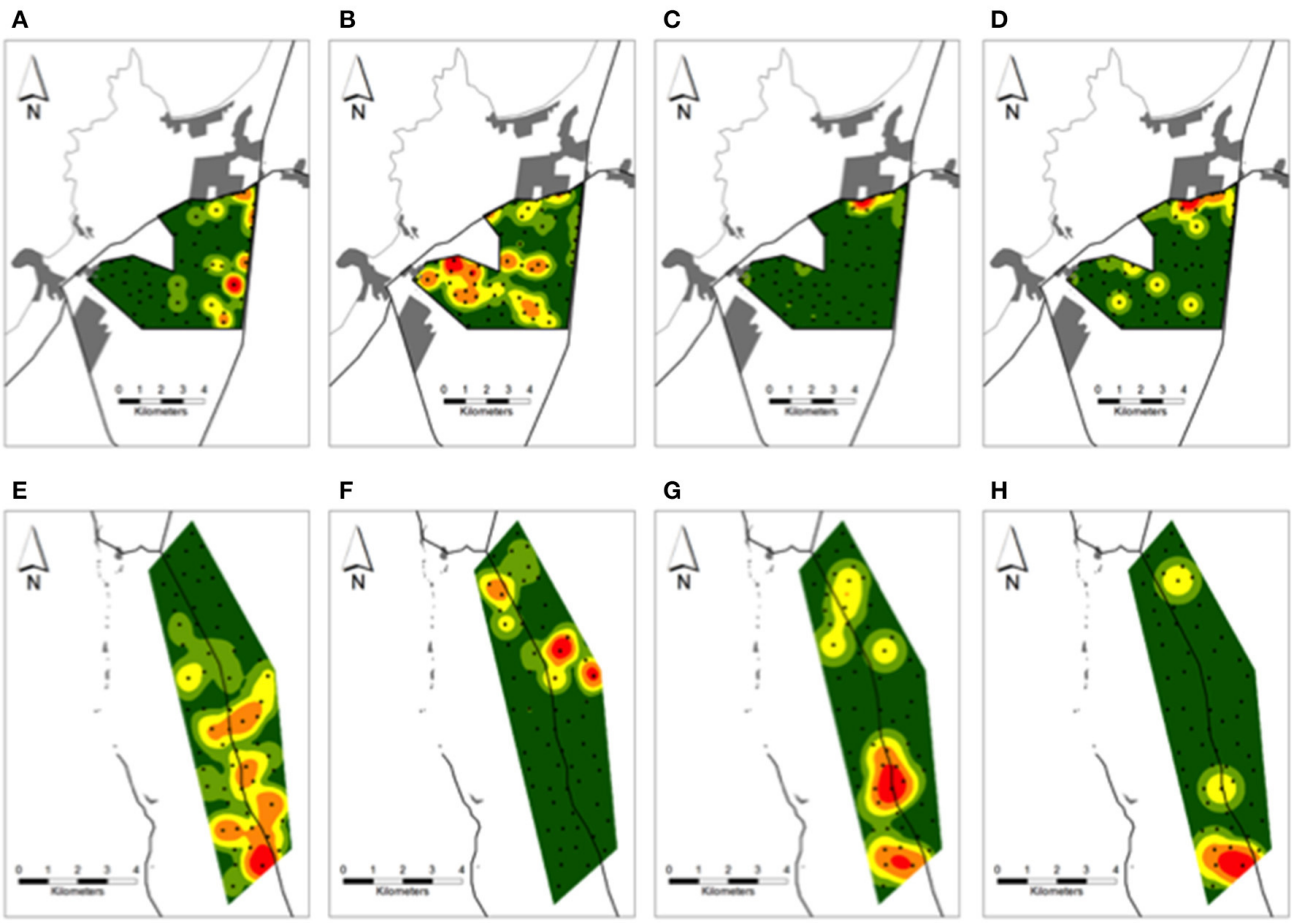
Kernel density surfaces of the number of **(A,E)** culpeos, **(B,F)** chillas, **(C,G)** dogs, and **(D)** dog-chilla fox and **(H)** dog-culpeo fox interactions over the periurban and rural sites in austral spring and summer between 2018 and 2020. Green to red color ramp represents lower (green) to higher (red) numbers of canid photos and interaction events per site-km^2^. Black circles over each site indicate camera trap stations. Proximate human settlements (gray polygons) and main roads (black lines) are drawn around each site. Results over the wild site are not shown.

In the periurban site, photos of culpeos were captured by 28% (17/60) of cameras, occurring as several hotspots nearby the southeast border of the site (Figure 4A). Chilla fox activity was documented by 90% (54/60) of cameras, expanding through the northern, central and southern sections on an east-west direction over the site (Figure 4B). Dogs were recorded by 43% (26/60) of cameras, exhibiting higher activity [68% (62/91) of dog photos] in the northeast border (Figure 4C). Dog activity hotspot overlapped with a zone where 65% (36/55) of dog-chilla interactions occurred, recorded by cameras nearby human settlements (range: 0.06–1.38 km) and main roads (range: 0.01–1.3 km) (Figure 4D). In the rural site, culpeos were recorded by 80% (48/60) of cameras, and their activity was concentrated from central toward southern portions of the site (Figure 4E). Chilla foxes [photographed by 58% (35/60) of cameras] conformed three hotspots in the northern half of the site (Figure 4F). Dogs were detected at 20% (12/60) of cameras and displayed higher activity [71% (10/14) of dog photos] in the south (Figure 4G). Thus, 71% (5/7) of dog-culpeo interactions were detected across cameras in the southern border (Figure 4H). In the wild site, culpeo fox was detected at 88% (53/60) of the cameras, showing higher occurrence along the central valleys of BFJNP. Overall dog activity was recorded by only three cameras, with 75% of dog visits (3/4) involved in the three interactions dog-culpeo fox we detected along the southeastern border of the site.

### Activity Patterns

**Figure 5:**
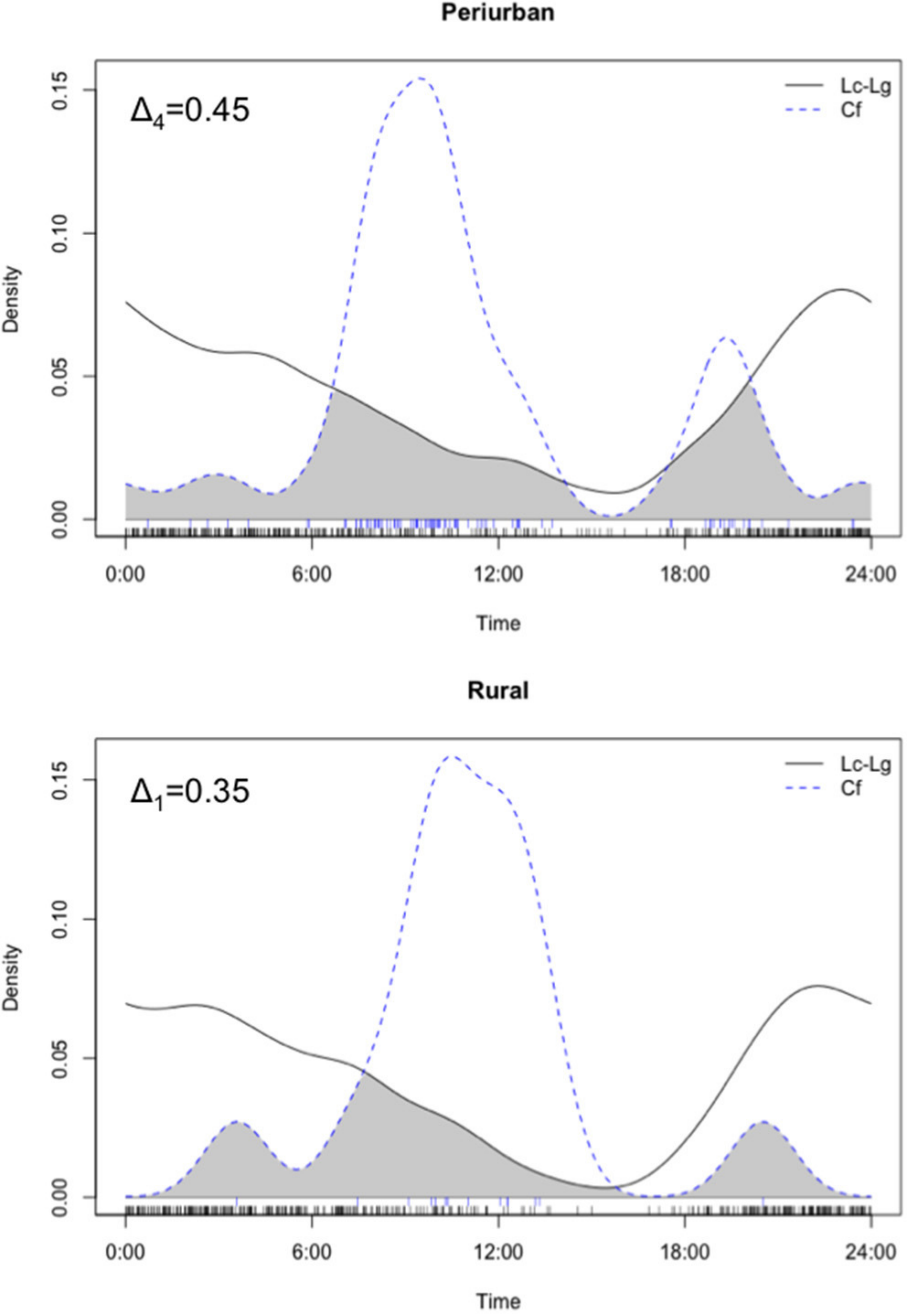
Density estimates of temporal activity displayed by culpeo (Lc) and chilla (Lg) foxes (solid line) and domestic dogs (Cf) (dashed line) in the periurban and rural sites in austral spring and summer between 2018 and 2020. Activity overlap between species is represented by the gray shaded area between lines. Deltas (Δ_4_ and Δ_1_) correspond to the coefficients of overlap. Fox activity is represented by combined photos of culpeo and chilla foxes. Data in the wild site are not graphed.

In overall, both culpeo and chilla were mainly crepuscular/nocturnal and exhibited prolonged nighttime activity (i.e., 77% of photos were distributed between 1,800 and 0600 h). Domestic dog activity peaked around 1,000 h, displaying a diurnal activity mostly concentrated between 0700 and 1,500 h (i.e., 72% of photos). We found high degree of activity overlap between both fox species in both periurban [Δ_1_ = 0.76 with 95% CI (0.64, 0.88)] and rural [Δ_4_ = 0.87 with 95% CI (0.81, 0.93)] sites. Thus, we pooled both fox species photos for further comparative analyses with dog temporal patterns. In overall, we found a low temporal activity overlap between dogs and foxes in the periurban (Δ_4_=0.45 with 95% CI 0.37, 0.53) and rural (Δ_1_=0.35 with 95% CI 0.19, 0.54) sites, and both groups exhibited significant differences in temporal patterns within both sites (periurban site: *U*^2^ = 2.47, *p* < 0.001; rural site: *U*^2^ = 0.60, *p* < 0.001) (Figure 5). Dog-fox temporal patterns consistently differed across sampling seasons (i.e., spring and summer) and years (i.e., year 1 and 2) within both sites (all Watson's two-sample *U*^2^-test had *p* < 0.01); thus, we only performed statistical comparisons between both canid groups' activity patterns at the site level. While we found a temporal activity overlap of Δ_1_ = 0.60 between dogs and culpeo foxes in the wild site, the coefficient precision was comparatively lower than the other sites (95% CI 0.31, 0.88), due to the smaller number of dog photos (*n* = 4).

### Predictors of Dog-Fox Interactions

Environmental predictors as site and distances to the nearest human settlement significantly predicted the number of interactions between dogs and foxes. Both variables were included in the top-ranked AICc model, which exhibited the highest AICc weight of evidence (*w*_i_ = 0.18). The cumulative AICc weight of evidence of these predictor variables was 0.48 across the five best-ranked candidate models (Table 3). Based on the top-ranked model, the rate of interactions (i.e., number of interactions/night-trap) between dogs and foxes in the periurban site was 10.58 times greater than in the wild site while holding all other variables constant; while rate of interactions in the rural site was not significantly different to the wild site (IRR 95% IC included 1) (Table 4). As distance to the nearest human facility showed a 1-km increase, the rate of interactions decreased by 37% while holding all other variables constant (Table 4). Although the other most supported models included distance to closest main road, year, season and NDVI, these variables did not significantly predict variations of interspecific interactions across field sites (IRR 95% IC included 1).

**Table 3:** Model selection for variables expected to predict the number of indirect interactions between dogs and foxes.

**Model**	***K*^**a**^**	**AICc**	**ΔAICc^**b**^**	**$w_{\text{i}}^{\text{c}}$**
Site + distance to human^d^ + distance to road^e^	7	351.7	0	0.18
Site + distance to human	6	353.2	1.53	0.08
Site + season + distance to human + distance to road	8	353.4	1.69	0.08
Site + year + distance to human + distance to road	8	353.7	1.98	0.07
Site + distance to human + distance to road + NDVI	8	353.7	2	0.07

a *Number of estimable model parameters*.

b *Difference between each model Akaike information criterion (AICc) value and the one of the lowest AIC (models with ΔAICc>2 are not shown)*.

^c^*Akaike weight of the model*.

d *Distance to closest human settlement*.

e *Distance to closest main road*.

**Table 4:** Estimates and incidence ratios (IRR) of predictor variables related to the number of indirect interactions between dogs and foxes.

	**Estimate (SE)**	**IRR**	**IRR 95% CI**	***z*-value**	**Pr (>|*z*|)**
Rural site^a^	1.06 (0.96)	3.45	(0.47–24.21)	1.70	ns
Periurban site^a^	2.12 (0.82)	10.58	(1.79–52.03)	3.54	***
Distance to human^b^	−0.68 (0.27)	0.63	(0.27–0.83)	−2.70	**
Distance to road^c^	−0.53 (0.29)	0.69	(0.31–1.02)	−2.07	*

*Parameters values of the best-ranked AICc model are presented*.

P-values: ns, p > 0.05;

* p < 0.05;

** p < 0.01;

*** *p < 0.001*.

a *Wild site defined as reference site*.

b *Distance to closest human settlement*.

c *Distance to closest main road*.

In overall, the time interval between dog and fox visits to a camera ranged from 3 h to 20 days (median = 3 days). Of the total dog-fox interaction events, 40% (29/72) exhibited ≤ 2 days between dog and fox visits (or vice versa), and 46% (26/57) of dog-chilla interactions were separated by this short-term time window. Median time interval between dog and chilla visits was shorter than between dog and culpeo visits at both the periurban and rural sites, ranging from 2.5 (dog-chilla) to 8.0 (dog-culpeo) (Table 2). We recorded nine occasions where dog and fox visits were photographed by the same camera within a 24-h diel period. In four out nine interactions (i.e., three dog-chilla and one dog-culpeo interactions), dog and fox photos were detected between 3 and 12 h apart across an entire daily cycle (between 0021 to 2,302 h). The type of interaction significantly predicted the time interval of dog-fox interactions, where the time interval of dog-culpeo interactions resulted greater than the time interval of dog-chilla interactions across all sites (*W* = 267, *p* = 0.03).

## Discussion

Our study constitutes the first effort to quantify the indirect interactions between domestic and wild canids by camera trapping in a wildlife-domestic interface in Chile. Our findings suggest that closeness to urbanized zones predicts the frequency of indirect interactions between domestic dogs and wild foxes, theoretically increasing the opportunities of exposure with persistently-shed and environmentally-resistant pathogens at sites co-occupied by both groups through an anthropization gradient. We found higher number of dog-fox interactions at a periurban site immediately adjacent to Tongoy and Guanaqueros suburbs, compared to other two more undisturbed sites increasingly distanced from towns. We showed that dogs interacted more frequently with chilla foxes (57 interactions) than with culpeo foxes (15 interactions), and the first interaction type occurred almost exclusively at the periurban site, where dogs and chillas were more frequently detected than in the other sites. Dog-chilla interactions resulted in shorter time intervals compared to dog-culpeo interactions, suggesting a higher potential risk of pathogen spillover between the first species pairing.

The higher rate of dog-fox interactions nearby Tongoy and Guanaqueros is likely mediated by the higher density, growth rate and turnover characterizing urban dog populations at the study area (32). Demographic factors as the higher human:dog ratio found in towns (1:4.1) compared to rural areas (1:1.7) (32), summed to dog subsidization by humans (20, 73, 74) and a higher frequency of free-roaming dogs with deficient veterinary care (20, 75), transform the urbanized zones into a source of multi-host pathogens threatening to wildlife. Furthermore, movement patterns as dogs displaying extra-territorial forays reaching up to 2 km away from their homes (60) and dog abandonment by people at roads adjacent to small towns and villages may have contributed to dog immigration into rural zones [e.g., (34, 76)]. In fact, solitary or grouped dogs were frequently sighted roaming along the northern periphery of the periurban site (F. Hernández pers. obs.), in agreement with the high dog detection rate (68% of dog photos) that defined the hotspot of dog-fox interactions we found around that zone.

Our findings corroborate previous observations about how dogs play a pivotal role in boosting contact rates with risk of pathogen spillover to wildlife through human-altered landscapes. For example, a study in Madagascar showed that the domestic dog is the most frequent invasive carnivore detected in closeness to human settlements within a protected area, where several carnivore species can compete for anthropogenic food resources and potentially transmit pathogens (42). In Australia, there is evidence of extensive spatial overlap between wild and domestic dogs along trails running across private and public managed lands, which may facilitate the spread of several indirectly transmitted pathogens, including zoonotic agents (41). In southern Chile, direct and indirect interactions among dogs, invasive American minks (*Neovison vison*) and river otters (*Lontra provocax*) were suggested to increase the risk of CDV exposure in proximity to rural villages and protected areas (24). However, the occurrence of interspecific contact events not necessarily has predicted pathogen transmission between domestic and native carnivores [e.g., (16, 17)], warranting further considerations about host- and environment-dependent factors that determine pathogen shedding and persistence as a measure of risk of spillover between species co-using same land use types.

Our findings about the relatively frequent interactions between dogs and chilla foxes at the periurban site aligned with the results reported by Acosta-Jamett et al. (35). By setting scent-stations, these authors found higher detection of chillas than culpeos surrounding the Tongoy-Guanaqueros urban periphery, suggesting that chillas were significantly more abundant in vicinity to human-altered landscapes, and consequently, prone to interact with domestic dogs. Chilla foxes are habitat generalists able to exploit a variety of habitats, including shrubby open habitats across lowland sites (77) and zones with intensive land use and disturbance (73, 78). The species is thought to tolerate moderate human presence and incidentally search for exotic mammals and refuse in vicinity of households (34, 73), creating ample opportunities of dog-fox interactions over habitat types preferred by both species (79). On the other hand, culpeo foxes seemed to have a weaker association to dogs-dominated habitats compared to chillas. While the wild site at the BFJNP constitutes a natural refuge for culpeos in the region (66), their detection rates decreased toward the periurban site, perhaps partially associated to a progressive increase in livestock predation resulting in retaliatory killing at human-dominated zones (66, 67).

Our research showed a low degree of overlap between temporal patterns exhibited by dogs and foxes, with a significant fine-scale time segregation detected between both groups within field site. This observed pattern is not surprising considering the typical behavioral avoidance displayed by subordinate species (wild foxes) to decrease encounters with dominant species (domestic dogs) within the context of interference competition, as it has been widely described for several carnivore guilds (41, 73, 79–81). Despite their marked temporal segregation, at a coarse spatial scale both canid groups seem to overlap on their habitat use (e.g., almost a third of the cameras visited by foxes were also visited by dogs at the periurban site), where dogs and foxes showed at least a 3-h shift between visits to the same camera station. In general, indirect interactions between dogs and chilla foxes were separated by a median of 2.5 days, a time window slightly higher than the estimated time of CDV survival in dry conditions (54). While a time window of 7.6 median days separated dog and culpeo visits across all sites, which largely corresponded to the ability for pathogens as CPV to survive for protracted periods (months or years) on feces (55). Considering the high to moderate CDV and CPV seroprevalences reported in dog (CDV: 67–69%; CPV: 83–94%) and fox (CDV: 43–50%; CPV: 29–83%) populations at the vicinity of Tongoy-Guanaqueros towns (35), perhaps interaction rates between dogs and foxes may facilitate exposure to these canid-borne viruses at the periurban zone. However, further studies are needed to understand whether effective cross-species pathogen transmission occurs in our system and disease agents largely jump from dogs to foxes [e.g., (82, 83)], or sympatric wild foxes would be able to maintain endemic pathogen infections, similarly to other wild carnivores elsewhere [e.g., (84, 85)].

Despite our study supported the usefulness of camera trapping for recording interactions between dogs and foxes, the actual role of indirect interaction rates as predictors of cross-species disease risk has to be interpreted with precaution. First, the lack of empirical studies on CDV and CPV survival times in natural environments prompted us to mostly rely on viral persistence under controlled experimental conditions (54, 55). Given our study was conducted in a semiarid ecosystem, we may have expected even more limited environmental pathogen longevity compared to other densely forested and humid regions (24, 42). However, patterns of long-lasting virus shedding by persistently infected hosts may also play a significant role in cross-species environmental exposure. Particularly, CDV shedding is thought to be limited up to 90 days post-infection mainly in oronasal exudates, but prolonged fecal shedding is not unexpected (86, 87), representing a source of pathogen transmission to carnivores co-using similar habitats [e.g., (88, 89)]. Second, due to our sampling design was composed by passive single-camera stations monitoring restricted areas during delimited periods, it prevented us from better describing social signaling behaviors potentially involved in disease transmission, such as sniffing, rolling, defecating, urinating. According to previous studies, cross-species scent marking could potentially promote the persistence and spread of pathogens released into feces and urine of dogs and foxes interacting at the wildlife-domestic interface [e.g., (24, 43, 90, 91)], which may be mediated by the interplay between prolonged shedding (e.g., CDV) and extended environmental resistance (e.g., CPV) characterizing several multi-host pathogens (92). Third, because random camera sampling could only record indirect interactions derived from animal movement through the landscape, perhaps the simultaneous monitoring of aggregation points (i.e., known canid paths) by camera trapping, and individual tracking with GPS telemetry or proximity loggers may be alternative approaches to account for more precise dog-fox interaction frequencies and their related temporal patterns (93).

Understanding the interplay between transforming landscapes and dynamic of interactions between invasive and native species is relevant to improve our overall comprehension of how human-driven land use changes predict the risk of pathogen spread at wildlife-domestic interfaces. Our study revealed that domestic dogs and free-ranging foxes (particularly chillas) interact indirectly across an anthropization gradient, concentrating their interaction events in close proximity to human settlements around the periphery of two coastal towns of the Coquimbo region. Based on the reported exposure to pathogens as CDV and CPV in dog and fox populations in the study area, we suggest this periurban zone may constitute a potential focus of pathogen exposure between both carnivore groups. Our study is the first systematic effort with camera traps designed to quantify interaction rates between domestic and wild canids in a wildlife-domestic interface in Chile. Beyond the epidemiological interest of the canine viruses we used as pathogen models, our indirect interaction framework may be applicable for several other pathogens environmentally transmitted between co-occurring domestic and wild canids such as tick or flea-borne diseases [e.g., (36, 38)]. For example, a recent study in Brazil suggested that domestic dogs can act as bridge-hosts of *Rickettsia parkeri*, agent of a tick-borne rickettsiosis in humans, by carrying the infected tick vector from habitats shared with wild fox species (the natural hosts) to houses of susceptible human populations (31). We expect our study will become a pivotal contribution to understand spatio-temporal patterns of interspecific contact between invasive and native carnivores within the context of multi-host pathogen dynamics. Our outcomes will inform theoretical epidemiological models designed to predict and minimize the contact risk between domestic and threatened species, and strengthen the potential to implement effective control strategies at the wildlife-domestic interface.

## Data Availability Statement

The raw data supporting the conclusions of this article will be made available by the authors, without undue reservation.

## Ethics Statement

Ethical review and approval was not required for the animal study because a non-invasive technique (i.e., camera traps) to estimate indirect interactions between domestic and wild canids was used.

## Author Contributions

FAH and GAJ conceived the ideas, designed the study, analyzed the data, and led the writing of the manuscript. All authors carried out the field work, collected the data, revised the manuscript, contributed critically to the drafts, and approved the final version for publication.

## Conflict of Interest

The authors declare that the research was conducted in the absence of any commercial or financial relationships that could be construed as a potential conflict of interest.
